# Individual Postprandial Glycemic Responses to Diet in n-of-1 Trials: Westlake N-of-1 Trials for Macronutrient Intake (WE-MACNUTR)

**DOI:** 10.1093/jn/nxab227

**Published:** 2021-07-13

**Authors:** Yue Ma, Yuanqing Fu, Yunyi Tian, Wanglong Gou, Zelei Miao, Min Yang, José M Ordovás, Ju-Sheng Zheng

**Affiliations:** Key Laboratory of Growth Regulation and Translational Research of Zhejiang Province, School of Life Sciences, Westlake University, Hangzhou, China; Westlake Intelligent Biomarker Discovery Lab, Westlake Laboratory of Life Sciences and Biomedicine, Hangzhou, China; Institute of Basic Medical Sciences, Westlake Institute for Advanced Study, Hangzhou, China; Key Laboratory of Growth Regulation and Translational Research of Zhejiang Province, School of Life Sciences, Westlake University, Hangzhou, China; Westlake Intelligent Biomarker Discovery Lab, Westlake Laboratory of Life Sciences and Biomedicine, Hangzhou, China; Institute of Basic Medical Sciences, Westlake Institute for Advanced Study, Hangzhou, China; Key Laboratory of Growth Regulation and Translational Research of Zhejiang Province, School of Life Sciences, Westlake University, Hangzhou, China; Westlake Intelligent Biomarker Discovery Lab, Westlake Laboratory of Life Sciences and Biomedicine, Hangzhou, China; Key Laboratory of Growth Regulation and Translational Research of Zhejiang Province, School of Life Sciences, Westlake University, Hangzhou, China; Westlake Intelligent Biomarker Discovery Lab, Westlake Laboratory of Life Sciences and Biomedicine, Hangzhou, China; Key Laboratory of Growth Regulation and Translational Research of Zhejiang Province, School of Life Sciences, Westlake University, Hangzhou, China; Westlake Intelligent Biomarker Discovery Lab, Westlake Laboratory of Life Sciences and Biomedicine, Hangzhou, China; Chronic Disease Research Institute, Department of Nutrition and Food Hygiene, Zhejiang University School of Public Health, Hangzhou, China; Jean Mayer USDA Human Nutrition Research Center on Aging at Tufts University, Boston, MA, USA; IMDEA Food Institute, Madrid, Spain; Key Laboratory of Growth Regulation and Translational Research of Zhejiang Province, School of Life Sciences, Westlake University, Hangzhou, China; Westlake Intelligent Biomarker Discovery Lab, Westlake Laboratory of Life Sciences and Biomedicine, Hangzhou, China; Institute of Basic Medical Sciences, Westlake Institute for Advanced Study, Hangzhou, China

**Keywords:** postprandial glycemic response, macronutrient, nutrition, n-of-1 trial, diet pattern

## Abstract

**Background:**

The role of different types and quantities of macronutrients on human health has been controversial, and the individual response to dietary macronutrient intake needs more investigation.

**Objectives:**

We aimed to use an ‘n-of-1’ study design to investigate the individual variability in postprandial glycemic response when eating diets with different macronutrient distributions among apparently healthy adults.

**Methods:**

Thirty apparently healthy young Chinese adults (women, 68%) aged between 22 and 34 y, with BMI between 17.2 and 31.9 kg/m^2^, were provided with high-fat, low-carbohydrate (HF-LC, 60–70% fat, 15–25% carbohydrate, 15% protein, of total energy) and low-fat, high-carbohydrate (LF-HC, 10–20% fat, 65–75% carbohydrate, 15% protein) diets, for 6 d wearing continuous glucose monitoring systems, respectively, in a randomized sequence, interspersed by a 6-d wash-out period. Three cycles were conducted. The primary outcomes were the differences of maximum postprandial glucose (MPG), mean amplitude of glycemic excursions (MAGE), and AUC_24_ between intervention periods of LF-HC and HF-LC diets. A Bayesian model was used to predict responders with the posterior probability of any 1 of the 3 outcomes reaching a clinically meaningful difference.

**Results:**

Twenty-eight participants were included in the analysis. Posterior probability of reaching a clinically meaningful difference of MPG (0.167 mmol/L), MAGE (0.072 mmol/L), and AUC_24_ (13.889 mmol/L·h) between LF-HC and HF-LC diets varied among participants, and those with posterior probability >80% were identified as high-carbohydrate responders (*n* = 9) or high-fat responders (*n* = 6). Analyses of the Bayesian-aggregated n-of-1 trials among all participants showed a relatively low posterior probability of reaching a clinically meaningful difference of the 3 outcomes between LF-HC and HF-LC diets.

**Conclusions:**

N-of-1 trials are feasible to characterize personal response to dietary intervention in young Chinese adults.

See corresponding editorial on page 2863.

## Introduction

Whether low-carbohydrate (LC) or low-fat (LF) diets are better for cardiometabolic health has been debated for decades, with inconsistent evidence from both observational studies and clinical trials (1, 2). These inconsistencies have been attributed primarily to differences in study designs, populations studied, and sample sizes. However, another important argument to consider is the substantial intra- and interperson variability in response to dietary factors (3–6). Several landmark studies have been conducted to characterize and predict the individual response to diet in humans, including an Israeli personalized nutrition cohort and the most recent personalized responses to dietary composition (PREDICT 1) study (7, 8), challenging the concept of ‘one-size-fits-all’ dietary recommendations. Although postprandial hyperglycemia has been associated with a higher risk of cardiometabolic diseases (9), postprandial glycemic response can be easily monitored by a wearable device and recovers to baseline within a short time, which makes it an ideal target for investigating the individual response to specific meals with n-of-1 clinical trials (7, 8, 10, 11).

In contrast to conventional randomized clinical trials focusing on group-level treatment differences, the n-of-1 clinical trial aims to identify individual responses to a given intervention in a controlled trial, which suits a chronic or frequently recurring condition and intervention with rapid onset and little carryover (11). Although this approach has mainly been applied in psychological and pharmaceutical fields (12, 13), some previous trials have used principles of n-of-1 research to design and/or interpret results in the nutritional field (14, 15). In fact, the intraindividual variability of glycemic responses has also been reported, and many statistical methods or machine-learning algorithms have been developed for personalized nutrition (7, 8). Additionally, in clinical practice, dieticians have been delivering personalized nutrition to some extent, accounting for each client's characteristics and clinical outcomes. With these signs of progress, the concept of n-of-1 trials is also becoming increasingly popular, supported and promoted by the International Collaborative Network for N-of-1 Clinical Trials and Single-Case Designs (16). Taken together, n-of-1 may hold great potential for its application in the nutrition field and provide a systematic approach toward personalized nutrition (17, 18).

Therefore, in the present study, we conducted a series of nutritional n-of-1 clinical trials to investigate individulized postprandial glycemic responses to different proportions of dietary fat and carbohydrate intake: the Westlake N-of-1 Trials for Macronutrient Intake (WE-MACNUTR). In the WE-MACNUTR, we aimed to predict individuals who responded better to a high-fat, low-carbohydrate (HF-LC) diet or to a low-fat, high-carbohydrate (LF-HC) diet, with regard to postprandial glycemic status. We examined glycemic responses by continuous glucose monitoring (CGM) data because multiple postprandial blood sample collections were challenging, which would place a considerable burden on the participants. The WE-MACNUTR also investigated the feasibility of n-of-1 clinical trials in the nutritional field, aiming to provide an example for future studies.

## Methods

### Study design and participants

The study was approved by the Ethics Committee of Westlake University (No. 20190919ZJS001) and registered at clinicaltrials.gov as NCT04125602. Participants were recruited from students and staff of Westlake University (Hangzhou, China) through an electronic poster and emails in October 2019, and all participants gave written informed consent. Inclusion criteria included adults aged between 18 and 65 y and willingness to join and complete the study. Exclusion criteria included: inability or unwillingness to provide informed consent; neurological conditions that might affect the assessment of the study measurement; hospitalization or surgery planned within 3 mo; gastrointestinal diseases; other severe medical conditions, such as liver, kidney, or systemic disease; pregnant or lactating women; tobacco, alcohol, or illicit drug abuse; antibiotics use within 2 wk before the trial; participants on a vegan diet; food allergy; no access to a smartphone or computer with an internet connection; those who could not speak Chinese; or concurrent participation in another intervention study.

The study was undertaken at Hangzhou, a city in southeast China, between 20 October, 2019, and 31 December, 2019. After screening and baseline data collection, 3 intervention sets comprising 4 6-d periods for each set were conducted. An n-of-1 trial would have to have >2 intervention pairs or cycles for a stable estimate of a treatment effect(19). Therefore, taking feasibility into account, we designed a 3-set ‘n-of-1 trial.’ Each participant completed 2 crossover experimental conditions within each study set with a wash-out period to reduce the potential carryover effect. The number of measurements within a treatment period was determined by the primary outcome (3 meals per day). Isocaloric diets were provided throughout the trial for each individual. According to a computer-generated randomization schedule, the order of HF-LC and LF-HC diets was randomly assigned within each of 3 pairs of interventions with a block size of 2. The order in which intervention was given was: LF-HC, HF-LC, HF-LC, LF-HC, HF-LC, and LF-HC (**Supplemental Figure 1**). Two research staff took charge of the recruitment and enrollment, and a team member not involved in the implementation of the trials carried out random sequence allocation.

A dietitian designed the diet for the intervention and the wash-out periods based on the Chinese Dietary Guidelines (2016) and Chinese Dietary Reference Intakes (2013) (20, 21). Men's and women's target energy intake was 2300 and 1900 kcal per day, respectively. The HF-LC diet comprised a 3-d diet consisting of 70% of total energy intake (E) from fat, 15%E from protein, and 15%E from carbohydrate (70% fat diet or 15% carbohydrate diet), whereas the other 3-d diet consisted of 60%E from fat, 15%E from protein, and 25%E from carbohydrate (60% fat diet or 25% carbohydrate diet). The LF-HC diet comprised a 3-d diet consisting of 20%E from fat, 15%E from protein, and 65%E from carbohydrate (20% fat diet or 65% carbohydrate diet), whereas the other 3-d diet consisted of 10%E from fat, 15%E from protein, and 75%E from carbohydrate (10% fat diet or 75% carbohydrate diet). The diet for the 6-d wash-out period consisted of 30% from fat, 15% from protein, and 55% from carbohydrate (30% fat diet or 55% carbohydrate diet). Detailed dietary macronutrient intake of the intervention and wash-out diet is provided in **Supplemental Table 1**. Recipes for the study meals during the intervention are also in **Supplemental Tables 2** and **3**.

Participants were instructed to consume only the provided foods or beverages. Participants consumed prepackaged and weighed meals (scheduled for: breakfast 07:00–09:00, lunch 11:00–13:00, dinner 17:00–19:00) for 72 d in total during the intervention. All participants were asked to complete a daily food checklist to assess their compliance. The sequentially numbered recipes of the study meals were provided to the chef at the Westlake University canteen, responsible for preparing the meals. Participants and researchers conducting the analyses were blinded to all randomization and packaging procedures until the completion of all analyses. A more detailed description of the WE-MACNUTR trial protocol is available elsewhere (22).

### Procedures

The enrolled participants spent the first 6-d period with a wash-out diet to obtain a relatively standardized baseline status before each intervention period. Prior to each intervention period, a continuous glucose monitoring system (CGMS; FreeStyle Libre Pro System, Abbott Diabetes Care) was inserted into the participant's subcutaneous fat tissue on the back of the upper arm, which was then secured with waterproof dressings. Following insertion, 1 h was used to allow the CGMS sensor to adjust to the interstitial fluid before the initial calibration. Every participant was required to wear the CGMS for CGM ≤8 d (CGM started 2 d before the intervention), covering each 6-d intervention period. The FreeStyle Libre Pro System consists of a sensor and a receiver/reader. The sensor is provided with a sterile catheter (0.4 mm caliper) inserted 5 mm under the skin and connected to a round disk (35 mm × 5 mm). The CV (11.75%) of 2 monitors on the same participant's arm showed the reliability of CGMs in the PREDICT 1 study (8). The reliability (R >0.80) of mean glucose was sufficient after 2 d of recording for individuals with normal glucose metabolism (23).

Participants were asked not to change their lifestyle or physical activity throughout the study period. Throughout each 6-d intervention period, they were asked to wear an AX3 band around the wrist (Axivity AX3 wrist-worn triaxial accelerometer, Open Lab, Newcastle University) to monitor their physical activity.

Participants had 12 visits to the study site, where their biological samples and related anthropometric data were collected (Supplemental Figure 1). During each visit, urine, feces, and saliva were collected for future metabolomics and microbiota profiling. Fasting venous blood was collected for biochemical tests during the 4 visits of the first study set. Participants were asked to complete daily a basic questionnaire using an online app to record their eating behaviors and health conditions, including emotional status, sleep duration, illness, and drug use.

### Biochemical and clinical chemistry analyses

Biochemical analyses were performed on fasting serum samples. Clinical chemistry analyses were performed with an ARCHITECT c16000 clinical chemistry analyzer (Abbott Laboratories) and Abbott reagents. The analytes were glucose (hexokinase), triglycerides (glycerophosphate oxidase), total cholesterol (cholesterol oxidase), LDL cholesterol (surfactant assay), HDL cholesterol (catalase activity assay), apoA1 (immunoturbidimetry), apoB (immunoturbidimetry), albumin (bromcresol green), creatinine (enzyme), aspartate transaminase (NAD), alanine transaminase (NAD), uric acid (uricase), and urea (urea enzyme). Insulin was measured in serum samples using an insulin ELISA kit (Jiangsu Jingmei Biological Technology Co., Ltd).

### Outcomes

The primary measurement was interstitial glucose concentrations measured by the CGMS every 15 min. The specific primary outcomes were maximum postprandial glucose (MPG) (24), mean amplitude of glycemic excursions (MAGE), and AUC_24_ (25, 26). MPG is the peak value of CGM within 3 h after the first bite of a meal or the maximum CGM value between 2 meals when the interval is <3 h. MAGE is obtained by measuring the arithmetic mean of the differences between consecutive peaks and nadirs provided that the differences are >1 SD around the mean glucose values. AUC_24_ refers to the total area under the CGM curve from 00:00 to 24:00 of the day. Other secondary outcomes, such as microbiome and urine metabolomic profiles will be analyzed and reported in the future.

### Statistical analysis

Data on baseline characteristics of study participants were expressed as mean ± SD or number (percentage). One-way ANOVA was used to analyze physical activity changes at the individual level based on the time of moderate and vigorous physical activity (27) recorded by AX3 bands.

We examined the effect of the LF-HC diet on postprandial blood glucose (PBG) concentrations compared with the HF-LC diet within each participant. Based on the previously reported difference of 3 mg/dL (0.167 mmol/L) in daytime MPG between young participants aged 25–45 y and those older than 45 y (69% participants were women) (28), we considered this magnitude of difference clinically meaningful. For MAGE, we considered the clinically meaningful difference as 1.3 mg/dL (0.072 mmol/L) based on the values derived from healthy participants aged 25–45 y versus those older than 45 y (28). In terms of AUC_24_, the threshold was 1.5 × 10^4^ mg/dL·min (13.9 mmol/L·h) based on values derived from young adults with normal and prediabetes glycated hemoglobin (HbA1c) (29). The MPG data from missing meals were not used. Regarding MAGE and AUC_24_, if an individual was absent from 3 intervention meals of a day, his/her corresponding data for the day were excluded.

A Bayesian analysis model with noninformative priors was applied to calculate the posterior probability of a clinically meaningful difference in MPG, MAGE, and AUC_24_ elicited by the different dietary patterns at the individual level (19). We estimated the posterior distributions of the parameters of interest using Markov chain Monte Carlo (MCMC) methods in Bayesian modeling. The Bayesian analysis differs from the usual frequentist approach (e.g. use of *P* values or CIs). Rather than focusing on the probability of different patterns in outcomes assuming specific treatment effects, Bayesian analysis calculates the probabilities of a treatment effect and expresses the uncertainty derived from finite data collection. Specifically, the observed outcome differences from the paired treatment periods for a given participant were combined into a mean difference that was assumed to follow a normal distribution centered about that participant's true mean effect difference μ_i_, and μ_i_ could be related to the treatments by linear regression such that μ_i_ = α+βx (where α was constant effects within a participant, β was treatment effects, x was the treatments with category values). To complete the model's Bayesian specification, prior distributions needed to be defined for α and β, and these prior informations described what was known about these parameters before the study. We chose normal distributions centered at 0 with large variance for α and β as noninformative prior distributions (30). The participant would be identified as a responder to a specific dietary pattern (HF-LC compared with the LF-HC diet) with respect to the outcome if the posterior probability of a meaningful intervention effect was >80%. Otherwise, the participant would be considered as a nonresponder (31). We defined responders and nonresponders by comparing the results of 2 interventions within an individual.

To explore the effects of dietary carbohydrate/fat ratios on the outcome measures with positive results by macronutrient categorization, we performed the Bayesian analysis model to estimate the mean difference and the posterior probability of reaching a meaningful difference (0.167 mmol/L for MPG, 0.072 mmol/L for MAGE, and 13.889 mmol/L·h for AUC_24_) for each participant, comparing the diets consisting of higher carbohydrate/lower fat proportion (LF-HC; 75% carbohydrate diet, 65% carbohydrate diet, and 25% carbohydrate diet) with those containing the lowest carbohydrate/highest fat proportion (HF-LC; 15% carbohydrate diet).

For the Bayesian hierarchical model meta-analysis at the population level, we performed a simulation-based statistical power calculation, referring to the method reported by Stunnenberg et al. (31). This simulation suggested that a sample size of 30 participants (individually, 3 sets per participant, 2 intervention periods per set, and 18 observations per intervention period) at group level would achieve a power of >99% to detect a prespecified meaningful difference (>0.167 mmol/L) in MPG, between diet interventions (HF-LC compared with the LF-HC diet), at a 5% 2-sided type I error level.

To generate an estimate of an intervention effect at the population level, we combined the n-of-1 results derived from participants with ≥1 completed intervention cycle. As our n-of-1 trials used multiple measurements over time on the same individual, a hierarchical Bayesian method could be used to combine the results from serials of n-of-1 trials and obtain posterior estimates of both population- and individual-level treatment effects. This methodology incorporates both random variation at the different levels, such as within-participant and between-participant variance (30). As described in the protocol (22), we specified a generalized linear mixed regression (adjusted for age, sex, and BMI), which constructed a separate regression model incorporating serial correlation that related longitudinal measurements for each participant, assuming a normal distribution of the measurements centered around each participant's true intervention effect. These regression models were then connected through a second-level random-effects model, which postulated that the individual-specific regression coefficients were related through a normal distribution centered around the population-level average coefficients. We had limited information about the variance parameters, and inverse γ-distributions were convenient computational choices for distributions of variance (32). Therefore, we chose a noninformative inverse γ prior distribution for the between-participant variance parameter. We chose 90%, rather than 80% (used in the above individual-level data analysis), as a threshold of posterior probability of a clinically meaningful difference in MPG, MAGE, and AUC_24_, as we found the Bayesian hierarchical model for the group level meta-analysis needs a higher threshold to reduce the type I error in the simulation-based statistical power calculation. In the Bayesian hierarchical model meta-analysis, we also performed the following sensitivity analyses for different types of missing data: *1*) analysis of all available data; *2*) analysis excluding participants who withdrew early from the trial; *3*) analysis of participants who completed the entire protocol, i.e. excluding those who withdrew early and those with valid data <50%; (4) analysis of individuals who completed the entire protocol with neither chronic diseases nor taking prescription medications.

All statistical analyses were done in R statistical (version 3.6.2), and the Bayesian analysis was performed using the *Open2BUGS* R package (33).

## Results

### Data analyzed

**FIGURE 1:**
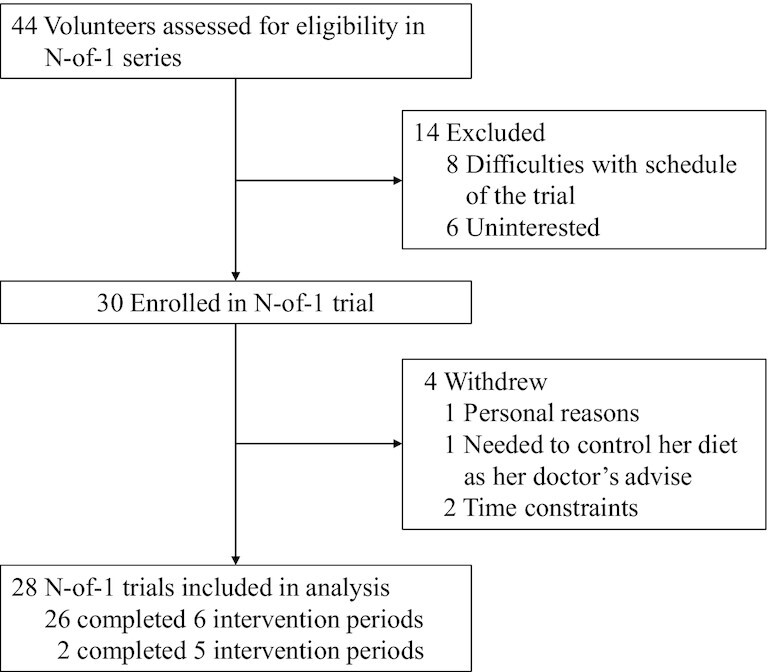
Study flow chart.

Of the 44 participants who contacted the study staff, 8 declined participation due to difficulties with the trial schedule, 6 were not interested in the trial, and eventually, 30 participants were enrolled. Twenty-six participants completed 6 intervention periods, 1 withdrew from the study for personal reasons after the data collection at baseline, 1 only completed the first intervention period due to her doctor's advice on diet control, and 2 underwent 5 periods and then quit the trial because of time constraints (**Figure 1**). Thus, data from 28 participants’ n-of-1 trials (82 LF-HC periods and 82 HF-LC periods) were analyzed at both individual and group levels. The mean adherence rate of the 28 participants was 98%, with the rate of a single person defined as the meals a person consumed divided by the total number of meals offered in the study. The mean proportion of meals with valid data in total intervention meals during individual participation was 90.2%, and the value was <50% for only 1 participant due to CGMS failure (**Supplemental Table 4**).

### Outcomes

The baseline characteristics of the participants are listed in **Supplemental Table 5**. Nine men and 19 women participants with a mean age of 26 y (SD, 2.8; range, 22–34 y) were enrolled and none of the participants had fasting glucose beyond 6.1 mmol/L. The AX3 band data showed no substantial physical activity changes during the intervention (**Supplemental Table 6**). No adverse effects were observed during the trials. As postprandial glycemia is an acute response to the meal, we did not assess carryover effects. All responders’ characteristics are shown in **Table 1**. The MPG, MAGE, and AUC_24_ of a single participant throughout the intervention periods are shown in **Supplemental Table 7** as an example of the change of these parameters in response to the interventions.

**TABLE 1:** Baseline characteristics of participants classified by response to the intervention^1^

Characteristic	HC-responders^2^ (*n* = 9)	HF-responders^2^ (*n* = 6)	Nonresponders (*n* = 13)	Overall (*n* = 28)
Age, y	24.0 (22.0–30.0)	26.0 (22.0–34.0)	26.0 (22.0–30.0)	26.0 (22.0–34.0)
Men, *n* (%)	2 (22)	3 (50)	4 (31)	9 (32)
BMI, kg/m^2^	20.8 (18.2–28.1)	22.2 (19.6–24.0)	22.8 (17.2–31.9)	22.0 (17.2–31.9)
Waist circumference, cm	74.0 (67.0–91.0)	80.0 (70.0–91.0)	79.0 (64.0–101)	78.5 (64.0–101)
Drinking, *No*. (%)				
Occasionally	2 (22)	5 (83)	9 (60)	16 (57)
Never	7 (78)	1 (17)	4 (40)	12 (43)
Fasting serum analyte				
Insulin, mU/L	26.3 (17.8–32.9)	24.3 (17.8–33.3)	24.8 (16.7–32.7)	25.5 (16.7–33.3)
Glucose, mmol/L	4.21 (4.00–5.02)	4.32 (3.88–4.46)	4.16 (3.58–4.56)	4.21 (3.58–5.02)
Triglycerides, mmol/L	0.54 (0.43–1.08)	0.76 (0.45–1.99)	0.69 (0.39–1.48)	0.65 (0.39–1.99)
Total cholesterol, mmol/L	4.45 (3.60–5.44)	4.64 (3.61–6.47)	4.08 (3.00–5.17)	4.38 (3.00–6.47)
LDL cholesterol, mmol/L	2.12 (1.26–2.77)	2.19 (1.15–3.57)	1.80 (1.12–2.67)	1.94 (1.12–3.57)
HDL cholesterol, mmol/L	1.73 (1.27–2.17)	1.49 (1.23–2.63)	1.68 (1.14–2.08)	1.64 (1.14–2.63)
ApoA1/apoB	1.92 (1.32–3.22)	1.55 (1.11–4.30)	2.10 (1.50–3.45)	2.05 (1.11–4.30)
Albumin, g/L	42.3 (39.4–43.2)	43.5 (42.6–45.7)	40.8 (39.2–47.0)	42.4 (39.2–47.0)
Creatinine, μmol/L	55.0 (46.0–70.0)	66.0 (54.0–76.0)	59.0 (46.0–76.0)	59.5 (46.0–76.0)
AST, U/L	15.0 (13.0–19.0)	17.5 (12.0–22.0)	14.0 (11.0–21.0)	15.0 (11.0–22.0)
ALT, U/L	11.0 (9.00–22.0)	15.0 (9.00–24.0)	9.00 (6.00–22.0)	10.5 (6.00–24.0)
Uric acid, μmol/L	276 (187–450)	280 (242–417)	282 (163–400)	280 (163–450)
Urea, mmol/L	3.70 (3.00–4.80)	3.95 (3.50–5.00)	4.00 (2.70–5.30)	3.80 (2.70–5.30)

1 Values are frequency (%) or median (range). ALT, alanine aminotransferase; AST, aspartate aminotransferase; HC, high carbohydrate; HF, high fat.

2 Responders for maximum postprandial glucose (MPG) or mean amplitude of glycemic excursions (MAGE).

**FIGURE 2:**
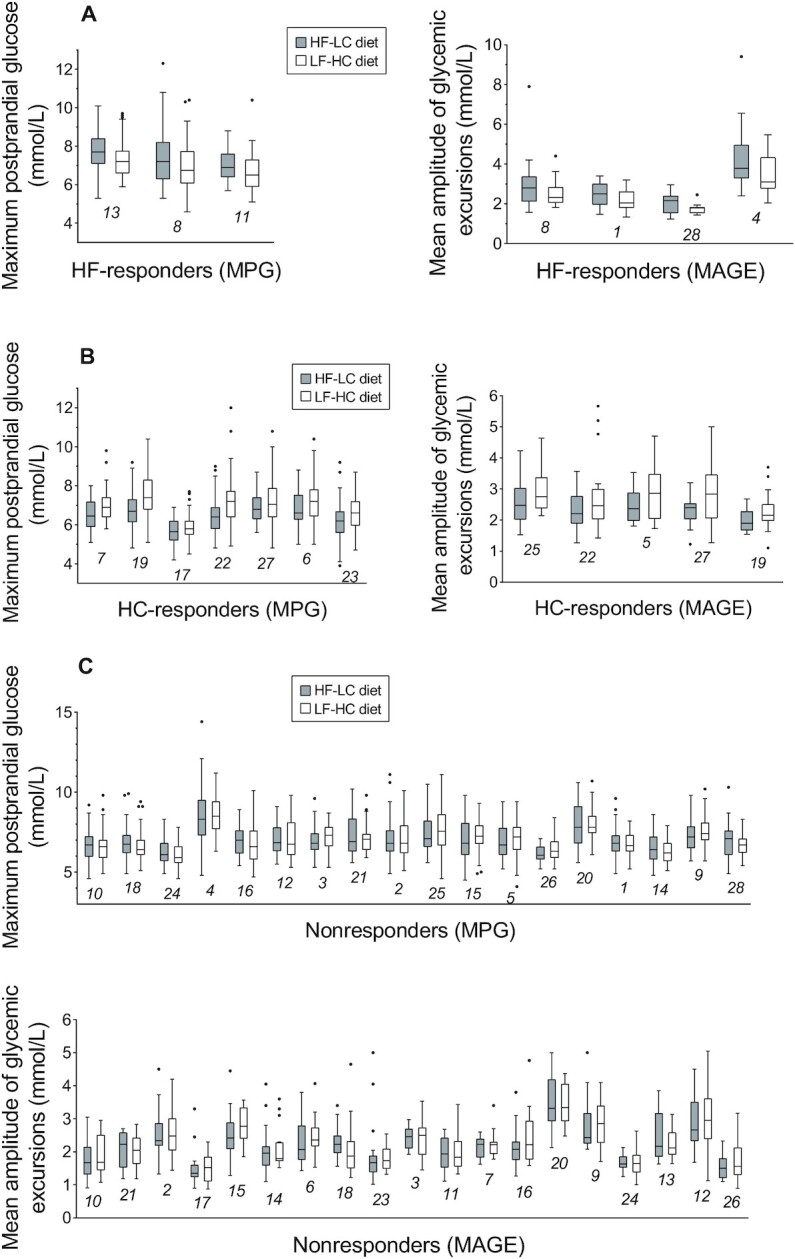
Individual-level maximum postprandial glucose and mean amplitude of glycemic excursions in participants during low-fat high-carbohydrate and high-fat low-carbohydrate interventions. Individual-level maximum postprandial glucose (MPG) or mean amplitude of glycemic excursions (MAGE) during 6 intervention periods (3 HF-LC periods illustrated by grey boxes and 3 LF-HC periods illustrated by white boxes) are separated on the *x*-axis, with participant numbers beneath data markers for crossreferencing with information in Table 2, Supplemental Tables 3, 5, and 6. Data are median (central line), IQR (box margins), adjacent values (whiskers), and outliers (dots). (A), HF-responders (MPG) (*n* = 3) and HF-responders (MAGE) (*n* = 4); (B), HC-responders (MPG) (*n* = 7) and HC-responders (MAGE) (*n* = 5); (C), nonresponders (MPG) (*n* = 18) and nonresponders (MAGE) (*n* = 19). The numbers in the figure, e.g. 13, 8, 11 in panel A, are participant numbers. HF-LC, high-fat low-carbohydrate; LF-HC, low-fat high-carbohydrate.

**FIGURE 3:**
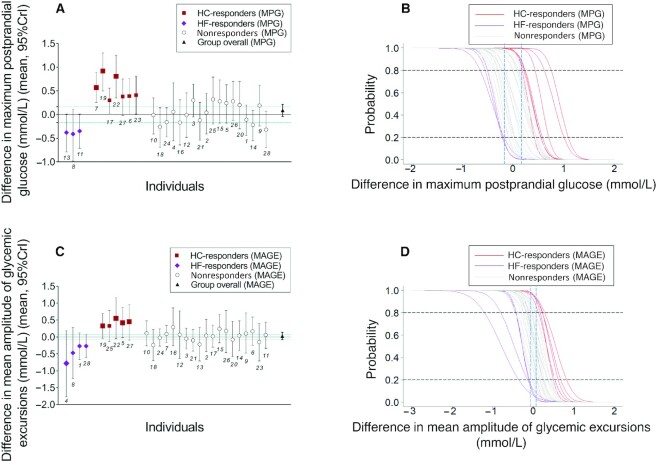
Results from Bayesian analysis of postprandial maximum glucose and mean amplitude of glycemic excursions at the individual and group level in young Chinese adults consuming low-fat high-carbohydrate compared with high-fat low-carbohydrate diet. (A) Mean difference between postprandial maximum glucose (MPG) from LF-HC and HF-LC diets and 95% credible interval (CrI) for each participant, *n* = 28. The estimates for the group are estimated using information from all participants. Individual-level effects are separated on the *x*-axis according to their response to LF-HC and HF-LC diets, with participant numbers beneath data markers for crossreferencing with information in Table 2, Supplemental Tables 3, 5, and 6. The size of the squares and rhombus is in direct proportion to the posterior probability of the difference of MPG between LF-HC and HF-LC periods higher than 0.167 mmol/L. Green dotted lines at *y* = 0.167 and –0.167 represent the threshold for a clinically meaningful effect. (B) Cumulative density function provides readout for the posterior probability (*y*-axis) belonging to the difference in MPG elicited by LF-HC and HF-LC diets (*x*-axis). The blue dashed lines provide readouts for posterior probabilities of reaching a clinically meaningful effect of 0.167 mmol/L difference. For HC-responders (MPG), *n* = 7, the posterior probability of difference in MPG elicited by LF-HC and HF-LC diets higher than 0.167 mmol/L is >80% (marked by the dashed line at *y* = 0.8). For HF-responders (MPG), *n* = 3, the corresponding posterior probability of difference in MPG higher than –0.167 mmol/L is lower than 20%, in other words, the posterior probability of difference in MPG elicited by HF-LC and LF-HC diets higher than 0.167 mmol/L is >80% in HF-responders (MPG). (C) Mean difference between mean amplitude of glycemic excursions (MAGE) from LF-HC and HF-LC diets and 95% CrI for each participant, *n* = 28. Green dotted lines at *y* = 0.072 and –0.072 represent the threshold for a clinically meaningful effect. (D) Cumulative density function provides readout for the posterior probability (*y*-axis) belonging to the difference between MAGE elicited by LF-HC and HF-LC diets (*x*-axis). The blue dashed lines provide readouts for posterior probabilities of reaching a clinically meaningful effect of 0.072 mmol/L difference. For HC-responders (MAGE), *n* = 5, the posterior probability of difference in MAGE elicited by LF-HC and HF-LC diets higher than 0.072 mmol/L is >80% (marked by the dashed line at *y* = 0.8). For HF-responders (MAGE), *n* = 4, the corresponding posterior probability of difference in MAGE higher than –0.072 mmol/L is lower than 20%, in other words, the posterior probability of difference in MAGE elicited by HF-LC and LF-HC diets higher than 0.072 mmol/L is >80% in HF-responders (MAGE). The numbers in the figure, e.g. 13, 8, 11 in panel A, are participant numbers. HF-LC, high-fat low-carbohydrate; LF-HC, low-fat high-carbohydrate.

For MPG, participants responded to LF-HC and HF-LC diets in 3 distinctive ways, which were defined as high-carbohydrate responders (HC-responders [MPG]), high-fat responders (HF-responders [MPG]), and nonresponders (MPG). A clinically meaningful MPG difference could be observed among 10 participants (**Figure 2**A–C), with the posterior probability of the difference reaching a clinically meaningful effect >80%. In 7 (HC-responders [MPG]) out of the 10 responders, the LF-HC diet increased MPG (the difference was >0.167 mmol/L) compared with the HF-LC diet, which was contrary to the other 3 individuals (HF-responders [MPG]) (**Figure 3**A, B; **Table 2**; **Supplemental Table 8**).

**TABLE 2:** Individual posterior probability of difference in postprandial blood glucose when participants consumed low-fat high-carbohydrate compared with high-fat low-carbohydrate diets^1^

	Posterior probability (%)					
	Difference of MPG (mmol/L)		Difference of MAGE (mmol/L)		Difference of AUC_24_ (mmol/L·h)	
Participant *No*.	<–0.167	>0.167	<–0.072	>0.072	<–13.889	>13.889
*1* ^2^	35.4	4.50	83.2	4.37	0	0
*2*	17.3	28.6	33.0	43.7	0	0
*3*	0.567	78.5	43.4	21.7	0	0.133
*4* ^2^	22.7	37.2	91.6	4.47	0	0
*5* ^3^	2.73	63.7	1.70	94.0	0.133	0
*6* ^3^	0.367	87.9	12.6	66.5	0	0
*7* ^3^	0	99.2	9.87	55.5	0	44.8
*8* ^2^	81.0	1.60	83.8	8.60	3.07	0
*9*	5.17	53.9	28.5	52.9	0	0
*10*	20.7	17.7	17.2	56.5	0	0
*11* ^2^	83.9	0.267	25.1	45.9	1.47	0
*12*	26.6	23.5	35.1	48.4	0	0
*13* ^2^	85.1	0.167	72.6	13.1	0.0333	0
*14*	63.0	0.600	32.5	43.0	0	0
*15*	2.73	69.4	6.87	78.3	0	0
*16*	51.7	7.90	10.5	77.5	24.8	0
*17* ^3^	0.0667	83.5	30.2	37.7	0	0
*18*	67.8	1.77	76.1	10.1	0	0
*19* ^3^	0	100	1.77	91.5	0	61.8
*20*	7.20	55.6	51.8	27.6	0	0
*21*	41.6	7.53	58.1	16.0	0	0
*22* ^3^	0	99.5	2.20	94.2	0	4.17
*23* ^3^	0.400	88.7	60.3	21.5	0	22.9
*24*	47. 7	1.70	34.7	23.1	0	0
*25* ^3^	3.13	73.0	4.43	86.4	0	0
*26*	1.33	70.7	19.6	64.3	0	0
*27* ^3^	0.433	86.3	3.03	91.7	0	0
*28* ^2^	77.5	0.800	86.8	2.67	2.87	0

1 Values are individual posterior probabilities (applied Bayesian analysis) of reaching a clinically meaningful difference in postprandial blood glucose between LF-HC and HF-LC diets, *n* = 28. This table reveals the posterior probability of the difference of outcomes between LF-HC and HF-LC periods reaching a clinically meaningful effect (MPG, 0.167 mmol/L; MAGE, 0.072 mmol/L; AUC_24_, 13.889 mmol/L). HF-LC, high-fat, low-carbohydrate; LF-HC, low-fat, high-carbohydrate; MAGE, mean amplitude of glycemic excursions; MPG, postprandial maximum glucose.

2 HF-responders for MPG or MAGE.

3 HC-responders for MPG or MAGE.

For MAGE, the difference of MAGE >0.072 mmol/L could be found in 9 participants, which were defined as responders, whereas the others are nonresponders. Higher MAGE was found in 5 participants (HC-responders [MAGE]) when eating an LF-HC diet, and in 4 participants (HF-responders [MAGE]) when eating an HF-LC diet (Figure 2A–C; Figure 3C, D; Table 2; Supplemental Table 8). Among these 9 individuals, 3 were HC-responders for both MAGE and MPG, and 1 participant was an HF-responder for both the above measures (Table 2; Supplemental Table 8). There was no noticeable, clinically meaningful difference for AUC_24_.

There was an upward trend in the mean difference between MPG elicited by diets with increased carbohydrate proportions for HC-responders. On the contrary, there was a downward trend for HF-responders (MPG) (**Supplemental Figure 2**A). The effect by macronutrient categorization could also be seen in the posterior probability for HC-responders (difference of MPG >0.167 mmol/L) (Supplemental Figure 2B) and HF-responders (difference of MPG < −0.167 mmol/L) (Supplemental Figure 2C). Similar trends of mean difference and posterior probability for MAGE are shown in Supplemental Figure 2D–F.

Analyses of the Bayesian-aggregated n-of-1 trials among all participants showed an 11.8%, 27.0%, and 0 posterior probability of reaching a clinically meaningful difference of MPG (>0.167 mmol/L or <–0.167 mmol/L), MAGE (>0.072 mmol/L or <–0.072 mmol/L), and AUC_24_ (>13.889 mmol/L·h or <–13.889 mmol/L) between LF-HC and HF-LC diets. Posterior probabilities of the intervention effects on MPG and MAGE at group level were higher in the sensitivity analysis but did not change the results substantially (**Supplemental Table 9**).

## Discussion

In the present feeding-based nutritional n-of-1 trials focusing on investigating an individual's postprandial glycemic response to different proportions of dietary fat and carbohydrate intake, we demonstrated the ability of this study design to predict responders or nonresponders to different ratios of macronutrient intake among young adults. With standardized interventions and strictly controlled eating behaviors, we identified not only HC-responders but also some HF-responders in terms of postprandial glucose response (PPGR). Such a strict feeding-based clinical trial, together with previous intervention- or observation-based precision nutrition studies (7, 8, 10), would provide mutually complementary evidence.

Biochemical individuality is more important than it is assumed to be because of its relation to susceptibility to human diseases (34). Postprandial glycemia has been implicated in the etiology of chronic metabolic diseases such as type 2 diabetes (T2D) and cardiovascular diseases (CVD) (9), which may militate against health via interstitial and cellular effects (35). Although a time difference (i.e. a “lag time”) exists for the equilibration of blood and interstitial glucose (36), interstitial glucose measured by CGMS reflects reliable physiological information on glucose in blood, interstitial space, and cells (37). The correlations of CGMS peak glucose and CV with reference glucose measurement (i.e. venous blood glucose concentrations) in a previous study were high (r = 0.89, r = 0.87 respectively) (38). In PREDICT 1, the correlation (r = 0.97) of participants’ incremental AUC in response to standardized meals also supports the reliability of the measurement with CGMS (8). Moreover, for the PREDICT 1 study, the variability of CGM was presented as the raw postprandial blood glucose trajectory, which visually showed high variability. In comparison, our results were based on integrated variables representing different scales of blood glucose variability over a period of time which were more stable and potentially clinically relevant.

All the above glucose components may contribute to the mechanisms that lead to diabetic and cardiovascular complications, including excessive protein glycation and activation of oxidative stress (9, 25). Hence, participants whose PBG was higher when eating a specific diet may need to modify their macronutrient distribution to reduce the potential health risks. If >1 aspect of MPG, MAGE, and AUC_24_ was high in response to a diet, for example, 4 participants’ MPG and MAGE were higher when eating 1 diet in the present study; the alternative diet might bring benefits for their metabolic health. We did not find responders for the AUC_24_, which might indicate AUC_24_ was less sensitive to the changes in dietary carbohydrate/fat ratio compared with the other variables (i.e. MPG and MAGE).

The effect of a low-carbohydrate or low-fat diet on glycemic control has been controversial (39, 40). In addition to the well-elucidated pathways of macronutrient metabolism, previous research also suggested that gastric emptying time might play a role in mediating the effects of macronutrients on postprandial glycemic responses. Although a 2-wk high-fat diet induced an accelerated gastric emptying over the linear phase of emptying, no perceptible changes in the pattern of emptying of the high-carbohydrate meal were detected (41). Therefore, gastric emptying time might take part in mediating the effects of different proportions of dietary fat and carbohydrate on postprandial glycemic variables (e.g. PMG, AUC_24_). Maintaining the same energy intake from protein between 2 interventions made it possible to compare substantial interindividual variation in postprandial glycemic response from interventions with different carbohydrate to fat ratios. Taking PPGR as an indicator, the present study, from a precision nutrition angle, provides a potential interpretation for these prior inconsistent findings about carbohydrate and glycemic control, in addition to the general explanation from the perspective of study design, sample size, or population ethnicities. Actually, individual variability in postprandial glucose in response to diet intake has been well recognized (6, 42). The PREDICT 1 study suggested that some individuals would experience large PBG excursions across most meals, whereas others would consistently experience modest responses (8). In the Israeli personalized nutrition study, interpersonal differences in the response of postprandial glucose to different meals were suggested to be predictable (7). In light of their results, our study integrated the concept of interpersonal variability of postprandial glucose response and the n-of-1 feeding trials to discover an optimal diet for each individual under a clinical trial setting.

Traditional investigation methodologies that make conclusions from a number of individuals to evaluate an average effect of interventions may lead to overlooking of variations in results between individuals (responders compared with nonresponders), and correspondingly may yield “null findings” in clinical studies (43), like the result on a group level in the present study. Thus, more robust study designs are needed, such as genotype-based randomized controlled trials (RCTs) and n-of-1 trials (3, 44). The present work, which predicted the optimal macronutrient distribution for an individual using objective data-driven criteria, provides information complementary to traditional trials and an example of applying the “n-of-1” study design in the nutrition field. The n-of-1 trial has great potential to help further understand the health effects of the complex interplay among genetics, microbiome, metabolism, food environment, and physical activity and then provide multidimensional and dynamic dietary advice tailored to an individual's unique characteristics (45). Nevertheless, it should be noted that the design allows flexibility for nutritional n-of-1 trials. Two common types of n-of-1 trials exist with either dynamic intervention or fixed intervention to a participant, and this study applied fixed interventions (11). The results of our n-of-1 trials without personalized interventions within the trial could be used to inform participants about their future dietary options. The design of the n-of-1 trial may also facilitate a detailed assessment about the sensitivity and specificity of an intervention, given that participants serve as their own controls in repeated crossover interventions, and all the analyses are based on personal outcomes for each individual (46). Furthermore, personal outcomes derived from the n-of-1 trial can be included in future clinical practice, which may enhance the clinical relevance of the treatment's effects. Finally, it is possible to combine the study design of the n-of-1 trial and observational study in a future investigation if we have a sufficient number of participants, which may maximize the strengths of both approaches.

In the present study setting, n-of-1 trials systematically offered an approach to personalized advice on choosing a diet based on one's unique postprandial glycemic response to macronutrient intake. Although it is generally considered that a low-carbohydrate dietmay help attenuate postprandial glucose increase, our n-of-1-based evidence identified 3 “outliers,” which showed the opposite response. These results indicate great potential for the application of n-of-1 trials in nutrition clinics, although more work needs to be done to make it more suitable for a clinical setting.

A significant strength is that we used a feeding n-of-1 clinical trial to investigate the personal PPGR with cutting-edge wearable devices monitoring continuous glucose values. Using our unique study design and statistical methods, we predicted responders and nonresponders at individual levels. Moreover, individualized nutritional decisions based on a series of randomized within-individual comparisons based on n-of-1 trials are likely to complement those based on traditional RCTs. A limitation of the present study is that we did not test postprandial insulin and could not elucidate the mechanism beyond the different responses to dietary macronutrient distribution, although we could identify the responders and nonresponders. A limited number of responders also prevented us from doing some group-level characterization or machine learning to identify the predictors. In addition, a potential carryover effect might affect the outcome. Although a 6-d wash-out period was applied between intervention periods, the parameters of interest during the wash-out interval may follow an asymptotic curve, and the observed changes in the parameters may depend on this state. Another limitation is that the present study is based on a group of young adults, consisting mainly of healthy young women, with limited generalizability to other age or ethnic groups. Moreover, we included some individuals with different baseline characteristics (e.g. chronic diseases) compared with others when we performed the hierarchical Bayesian analysis at the population level, which may bias our group-level results. Nevertheless, the personal characteristics were unlikely to bias our primary outcomes, which demonstrated the individual's postprandial glycemic response to diets, with each participant acting as her/his own control. In addition, due to the complexity of the Chinese diets and cooking methods, there is some deviation between our targeted macronutrient intakes and the delivered ones. Using our study as a model, researchers may potentially develop a more concise and straightforward strategy to implement the nutritional n-of-1 trial into daily clinical practice in the future.

In conclusion, our study, with a novel n-of-1 trial design, indicates that young participants without diabetes show substantial interindividual variation in postprandial glycemic response to HF-LC and LF-HC diets, and we identified specific HC-responders and HF-responders after the intervention. The present study suggests that the n-of-1 clinical trial can be a feasible study design in nutritional research to precisely characterize the personal response to specific dietary or nutritional intervention. More nutritional n-of-1 trials are warranted in the future as an important component in the precision nutrition field.

## Supplementary Material

nxab227_Supplemental_File

## Data Availability

The deidentified individual participant data will be made available after publication. Requests on data sharing can be made by contacting the corresponding author and proposals are required to be attached for approval.

## Notes

This study was supported by the National Natural Science Foundation of China (82073529, 81903316), the Zhejiang Ten-thousand Talents Program (2019R52039), the Zhejiang Provincial Natural Science Foundation of China (LQ21H260002), the China Postdoctoral Science Foundation (2020M681945), and the Zhejiang Postdoctoral Science Foundation (ZJ2020076). The funders of the study had no role in study design, data collection, data analysis, data interpretation, or writing of the manuscript. The corresponding author had full access to all the data in the study and had full responsibility for the decision to submit it for publication.

Author disclosures: The authors report no conflicts of interest.

Supplemental Figures 1–2 and Supplemental Tables 1–9 are available from the “Supplementary data” link in the online posting of the article and from the same link in the online table of contents at https://academic.oup.com/jn/.

YM, YF, and YT contributed equally to the work.

Abbreviations used: WE-MACNUTR, Westlake N-of-1 Trials for Macronutrient Intake; HF-LC, high-fat, low-carbohydrate; LF-HC, low-fat, high-carbohydrate; MPG, maximum postprandial glucose; PBG, postprandial blood glucose; MAGE, mean amplitude of glycemic excursions; CGMS, continuous glucose monitoring systems system; CGM, continuous glucose monitoring; PPGR, postprandial glucose response; HC-responder, high-carbohydrate responder; HF-responder, high-fat responder; PREDICT, personalized responses to dietary composition; RCT, randomized controlled trial.
