# Supplementary material for: Individual Postprandial Glycemic Responses to Diet in n-of-1 Trials: Westlake N-of-1 Trials for Macronutrient Intake (WE-MACNUTR)
Source: J Nutr. 2021 Jul 13;151(10):3158–67. doi: 10.1093/jn/nxab227 (PMC8485912; doi:10.1093/jn/nxab227)
Supplement: nxab227_Supplemental_File [file nxab227_supplemental_file.docx]

**Online supplemental data**

**Supplemental Table 1. Daily dietary intake breakdown of both diet arms (HF-LC and LF-HC) and the wash-out diet provided to participants**^1^

|  | HF-LC diet (*n* = 28) | | | | LF-HC diet (*n* = 28) | | | | Wash-out diet (*n* = 28) | |
| --- | --- | --- | --- | --- | --- | --- | --- | --- | --- | --- |
|  | 70% Fat diet | | 60% Fat diet | | 20% Fat diet | | 10% Fat diet | | 30% Fat diet | |
|  | Men (n=9) | Women (n=19) | Men (n=9) | Women (n=19) | Men (n=9) | Women (n=19) | Men (n=9) | Women (n=19) | Men (n=9) | Women (n=19) |
| Carbohydrate (g) | 147±1.99 | 118±4.04 | 173±4.95 | 142±5.10 | 344±1.10 | 289±1.11 | 350±5.27 | 303±4.12 | 298±7.97 | 241±7.52 |
| Carbohydrate (%E) | 25.5±0.33 | 24.3±1.02 | 29.3±1.26 | 28.9±1.54 | 60.0±0.54 | 60.6±0.78 | 61.6±0.55 | 63.7±0.80 | 52.2±1.36 | 50.7±2.08 |
| Glycemic index | 199±9.01 | 196±9.39 | 179±8.33 | 173±5.13 | 183±5.45 | 177±4.89 | 186±17.3 | 181±15.7 | 199±9.14 | 187±14.0 |
| Glycemic load | 90.4±4.42 | 74.2±3.69 | 95.6±1.85 | 75.9±0.26 | 194±3.71 | 154±6.77 | 201±22.8 | 165±21.6 | 245±19.5 | 276±24.2 |
| Fiber (g) | 8.97±1.40 | 7.66±1.10 | 11.6±1.23 | 10.1±0.92 | 19.7±3.00 | 17.2±2.88 | 15.9±3.48 | 14.8±3.16 | 14.4±3.93 | 12.7±3.66 |
| Protein (g) | 99.7±10.8 | 86.5±6.71 | 106±2.65 | 90.1±2.50 | 88.5±4.48 | 71.4±3.49 | 79.5±1.46 | 66.1±2.92 | 92.9±7.42 | 80.6±10.5 |
| Protein (%E) | 17.3±1.87 | 17.9±1.27 | 18.1±0.98 | 18.4±1.18 | 15.4±0.70 | 15.0±0.62 | 14.0±0.54 | 13.9±0.61 | 16.3±1.35 | 16.9±1.77 |
| Fat (g) | 147±3.97 | 125±1.24 | 138±9.65 | 115±9.85 | 62.8±0.76 | 51.7±1.39 | 61.8±3.98 | 47.4±1.43 | 79.9±5.52 | 68.5±4.81 |
| Fat (%E) | 57.3±1.55 | 57.9±0.42 | 52.6±1.98 | 52.7±2.54 | 24.6±0.32 | 24.4±0.41 | 24.4±1.03 | 22.4±0.71 | 31.5±1.92 | 32.4±1.97 |
| Saturated fat (g) | 27.8±1.75 | 24.1±1.56 | 25.8±3.14 | 22.1±2.92 | 10.4±0.37 | 9.00±0.83 | 11.3±1.38 | 8.91±0.98 | 19.5±3.09 | 17.1±2.82 |
| Monounsaturated fat (g) | 37.3±3.89 | 32.4±3.50 | 34.5±4.48 | 29.1±4.05 | 13.0±2.07 | 11.1±0.65 | 14.6±2.34 | 11.1±1.56 | 27.2±7.78 | 23.2±7.20 |
| Polyunsaturated fat (g) | 51.9±2.58 | 44.8±1.80 | 46.8±5.35 | 37.9±3.98 | 14.0±1.12 | 12.9±2.02 | 19.3±0.83 | 14.5±1.69 | 16.8±1.90 | 14.0±1.63 |

^1^Values are means ± SDs. The high-fat, low-carbohydrate diet consisted of a 3-day diet of 60% of total energy intake(E) from fat, 25%E from carbohydrate, and 15%E from protein (60% fat diet) and 3-day diet of 70%E from fat, 15%E from carbohydrate, and 15%E from protein (70% fat diet). The low-fat, high-carbohydrate diet consisted of a 3-day diet of 20%E from fat, 65%E from carbohydrate, and 15%E from protein (20% fat diet) and a 3-day diet of 10%E from fat, 75%E from carbohydrate, and 15%E from protein (10% fat diet). An isocaloric wash-out diet comprising 30%E from fat, 55% from CHO, and 15% from protein (30% fat diet). Total daily estimated energy intake was calculated 2300 kcal/d and 1900 kcal/d for men and women, respectively. HF-LC, high fat, low carbohydrate; LF-HC, low fat, high carbohydrate.

**Supplemental Table 2. One-day sample menu of intervention set 1 of n-of-1 trials for men**^1^

| Meal | HF-LC diet | | | LF-HC diet | | | Wash-out diet | | |
| --- | --- | --- | --- | --- | --- | --- | --- | --- | --- |
|  | Food item | | Weight | Food item | | Weight | Food item | | Weight |
| Breakfast | Dish A | Millet porridge | 120g | Dish A | Millet porridge | 310g | Dish A | Rice porridge | 250g |
|  | Dish B | Deep-fried dough sticks | 70.0g | Dish B | Deep-fried dough sticks | 40.0g | Dish B | Wheat pancake | 100g |
|  | Dish C | Bread | 30.0g | Dish C | Bread | 100g | Dish C | Fried dumpling (Bok choi and pork) | 32.0g |
|  | Dish D | Fried egg | 80.0g | Dish D | Boiled egg | 55.0g | Dish D | Fried egg | 55.0g |
|  |  |  |  |  |  |  | Fruit | Apple | 230g |
|  | Soybean oil |  | 10.0g | Soybean oil |  | 5.00g | Soybean oil |  | 14.0g |
| Lunch | Rice |  | 100g | Rice |  | 210g | Rice |  | 210g |
|  | Dish A | Lettuce with pork | 170g | Dish A | Lettuce with pork | 150g | Dish A | Sauteed potatoes with green pepper and eggplants | 265g |
|  | Dish B | Fried peeper and potato | 90.0g | Dish B | Fried peeper and potato | 280g | Dish B | Lean pork with king oyster mushroom | 140g |
|  | Dish C | Bass | 120g | Dish C | Bass | 100g | Dish C | Sole fish | 110g |
|  | Fruit | Orange | 70.0g | Fruit | Orange | 270g |  |  |  |
|  | Nut | Walnut | 40.0g | Nut | Walnut | 15.0g | Nut | Almond | 20.0g |
|  | Soybean oil |  | 20.0g | Soybean oil |  | 7.50g | Soybean oil |  | 10.0g |
| Dinner | Rice |  | 100g | Rice |  | 210g | Rice |  | 210g |
|  | Dish A | Chicken leg | 150g | Dish A | Chicken leg | 50.0g | Dish A | Pak choi | 130g |
|  | Dish B | Ham with wax gourd | 165g | Dish B | Ham with wax gourd | 140g | Dish B | Pork rib with cowpea | 180g |
|  | Dish C | Baby cabbage | 90.0g | Dish C | Baby cabbage | 140g | Dish C | Beef tendon | 180g |
|  | Fruit | Banana | 50.0g | Fruit | Banana | 230g |  |  |  |
|  |  |  |  |  |  |  | Milk |  | 250ml |
|  | Soybean oil |  | 20.0g | Soybean oil |  | 7.50g | Soybean oil |  | 10.0g |

^1^Values are weight of foods. Two intervention diets contained a variety of foods from main food groups, including vegetables and fruits, starchy products, meat and alternatives as well as milk and alternatives. The breakfast of HF-LC and LF-HC diets included four dishes, one type of porridge or soybean milk, two types of starchy food such as buns or pancakes, and eggs. Both lunch and dinner have three different dishes served with rice for two intervention groups. Fresh fruits, nuts and milk were provided to help participants meet their needs for vitamins, minerals and other nutrients as well as the total energy requirements. The menus for HF-LC and LF-HC diets were almost identical, apart from the detailed macronutrient contents. In total of 18 menus were provided for each intervention diet within 6 days, which added up 36 food patterns throughout the intervention period. The wash-out diet was designed with the same calorie as intervention diets but with different food items in order to provide the participants a more balanced and diverse diet. Therefore, having the amount and types of food provided contributes to the overall health and vitality. Participants *n* = 28. HF-LC, high fat, low carbohydrate; LF-HC, low fat, high carbohydrate.

**Supplemental Table 3. One-day sample menu of intervention set 1 of n-of-1 trials for women**^1^

| Meal | HF-LC diet | | | LF-HC diet | | | Wash-out diet | | |
| --- | --- | --- | --- | --- | --- | --- | --- | --- | --- |
|  | Food item | | Weight | Food item | | Weight | Food item | | Weight |
| Breakfast | Dish A | Millet porridge | 120g | Dish A | Millet porridge | 310g | Dish A | Rice porridge | 250g |
|  | Dish B | Deep-fried dough sticks | 60.0g | Dish B | Deep-fried dough sticks | 30.0g | Dish B | Wheat pancake | 85.0g |
|  | Dish C | Bread | 15.0g | Dish C | Bread | 100g | Dish C | Fried dumpling (Bok choi and pork) | 16.0g |
|  | Dish D | Fried egg | 80g | Dish D | Boiled egg | 50.0g | Dish D | Fried egg | 50.0g |
|  |  |  |  |  |  |  | Fruit | Apple | 230g |
|  | Soybean oil |  | 9.00g | Soybean oil |  | 4.00g | Soybean oil |  | 12.0g |
| Lunch | Rice |  | 80.0g | Rice |  | 150g | Rice |  | 120g |
|  | Dish A | Lettuce with pork | 130g | Dish A | Lettuce with pork | 145g | Dish A | Sauteed potatoes with green pepper and eggplants | 245g |
|  | Dish B | Fried peeper and potato | 65.0g | Dish B | Fried peeper and potato | 275g | Dish B | Lean pork with king oyster mushroom | 120g |
|  | Dish C | Bass | 100g | Dish C | Bass | 80.0g | Dish C | Sole fish | 100g |
|  | Fruit | Orange | 70.0g | Fruit | Orange | 270g |  |  |  |
|  | Nut | Walnut | 35.0g | Nut | Walnut | 15.0g | Nut | Almond | 10.0g |
|  | Soybean oil |  | 20.0g | Soybean oil |  | 5.00g | Soybean oil |  | 7.50g |
| Dinner | Rice |  | 90.0g | Rice |  | 145g | Rice |  | 130g |
|  | Dish A | Chicken leg | 120g | Dish A | Chicken leg | 40.0g | Dish A | Pak choi | 120g |
|  | Dish B | Ham with wax gourd | 140g | Dish B | Ham with wax gourd | 128g | Dish B | Pork rib with cowpea | 170g |
|  | Dish C | Baby cabbage | 80.0g | Dish C | Baby cabbage | 140g | Dish C | Beef tendon | 180g |
|  | Fruit | Banana | 50.0g | Fruit | Banana | 230g |  |  |  |
|  |  |  |  |  |  |  | Milk |  | 250ml |
|  | Soybean oil |  | 20.0g | Soybean oil |  | 5.00g | Soybean oil |  | 7.50g |

^1^Values are weight of foods. Two intervention diets contained a variety of foods from main food groups, including vegetables and fruits, starchy products, meat and alternatives as well as milk and alternatives. The breakfast of HF-LC and LF-HC diets included four dishes, one type of porridge or soybean milk, two types of starchy food such as buns or pancakes, and eggs. Both lunch and dinner have three different dishes served with rice for two intervention groups. Fresh fruits, nuts and milk were provided to help participants meet their needs for vitamins, minerals and other nutrients as well as the total energy requirements. The menus for HF-LC and LF-HC diets were almost identical, apart from the detailed macronutrient contents. In total of 18 menus were provided for each intervention diet within 6 days, which added up 36 food patterns throughout the intervention period. The wash-out diet was designed with the same calorie as intervention diets but with different food items in order to provide the participants a more balanced and diverse diet. Therefore, having the amount and types of food provided contributes to the overall health and vitality. Participants *n* = 28. HF-LC, high fat, low carbohydrate; LF-HC, low fat, high carbohydrate.

**Supplemental Table 4. Participant characteristics and meals with valid data**^1^

| Participant | Age (y) | Sex | BMI^2^ (kg/m^2^) | Meals with valid data^3^, *No.* (%) (%) |
| --- | --- | --- | --- | --- |
| *1* | 26 | male | 22.2 | 102 (94.4) |
| *2* | 22 | female | 18.1 | 108 (100) |
| *3* | 26 | male | 22.8 | 96 (88.9) |
| *4* | 34 | male | 22.1 | 93 (86.1) |
| *5* | 25 | female | 19.7 | 105 (97.2) |
| *6* | 22 | female | 20.3 | 102 (94.4) |
| *7* | 22 | male | 28.1 | 92 (85.2) |
| *8* | 22 | female | 20.5 | 105 (97.2) |
| *9*^4^ | 26 | female | 31.9 | 89 (98.9) |
| *10* | 24 | female | 20.1 | 107 (99.1) |
| *11* | 25 | female | 19.6 | 90 (83.3) |
| *12* | 30 | female | 19.4 | 88 (81.5) |
| *13* | 26 | female | 24.0 | 97 (89.8) |
| *14* | 25 | female | 23.3 | 100 (92.6) |
| *15* | 28 | male | 22.9 | 96 (88.9) |
| *16* | 24 | female | 21.9 | 94 (87.0) |
| *17* | 23 | female | 21.4 | 101 (93.5) |
| *18* | 28 | female | 22.3 | 101 (93.5) |
| *19* | 23 | male | 23.9 | 104 (96.3) |
| *20* | 27 | male | 24.5 | 80 (74.1) |
| *21* | 28 | female | 17.2 | 108 (100) |
| *22* | 30 | female | 21.2 | 97 (89.8) |
| *23* | 30 | female | 20.8 | 92 (85.2) |
| *24* | 23 | male | 23.5 | 98 (90.7) |
| *25* | 26 | female | 18.2 | 108 (100) |
| *26* | 25 | female | 24.8 | 51 (47.2) |
| *27* | 24 | female | 20.3 | 99 (91.7) |
| *28*^4^ | 27 | male | 23.3 | 88 (97.8) |

^1^Participants *n* = 28.

^2^BMI (body mass index) was measured at baseline.

^3^Values are the numbers of meals (percentage). The percentage refers to the proportion of meals with valid data in total intervention meals during individual participation. Absence from intervention meals and CGMS failure accounted for invalid or missing data.

^4^The participant completed 5 intervention periods.

**Supplemental Table 5. Baseline** **Participant Characteristics**^1^

| Characteristics | Men (*n* = 9) | Women (*n* = 19) |
| --- | --- | --- |
| Age, y | 26.0 (21.0-34.0) | 25.0 (22.0-30.0) |
| BMI, kg/m^2^ | 23.3 (22.1-28.1) | 20.5 (17.2-31.9) |
| Waist circumference, cm | 87.0 (79.0-91.0) | 73.5 (64.0-101) |
| Chronic diseases, *n* (%) | 2 (22)^2^ | 3 (15)^3^ |
| Dietary supplement intake, *n* (%) | 0 | 1(5)^4^ |
| Drinking, *n* (%) |  |  |
| Occasionally | 6 (67) | 10 (53) |
| Never | 3 (33) | 9 (47) |
| Fasting serum analyte |  |  |
| Insulin, mU/L | 33.3 (17.4-62.4) | 33.1 (16.7-61.9) |
| Fasting serum glucose, mmol/L | 4.15 (3.88-4.56) | 4.24 (3.58-5.02) |
| Triglycerides, mmol/L | 0.95 (0.43-1.99) | 0.57 (0.39-0.92) |
| Total cholesterol, mmol/L | 4.45 (3.00-6.47) | 4.30 (3.44-5.44) |
| LDL cholesterol, mmol/L | 2.32 (1.12-3.57) | 1.86 (1.12-2.77) |
| HDL cholesterol, mmol/L | 1.50 (1.19-1.94) | 1.73 (1.14-2.63) |
| ApoA1/ApoB | 1.51 (1.11-3.22) | 2.10 (1.50-4.30) |
| Albumin, g/L | 42.6 (39.4-47.0) | 42.3 (39.2-46.7) |
| Creatinine, μmol/L | 70.0 (66.0-76.0) | 55.0 (46.0-64.0) |
| AST, U/L | 17.0 (13.0-21.0) | 14.0 (11.0-22.0) |
| ALT, U/L | 20.0 (7.00-24.0) | 10.0 (6.00-24.0) |
| Uric acid, μmol/L | 352 (278-450) | 248 (163-316) |
| Urea, mmol/L | 4.60 (3.70-5.00) | 3.80 (2.70-5.30) |

^1^Values are frequency (percent) or median [range]. None of the participants were smokers. ALT, alanine aminotransferase; Apo, Apolipoprotein; AST, aspartate aminotransferase; BMI, body mass index; HDL, high-density lipoprotein; LDL, low-density lipoprotein.

^2^Chronic tonsillitis, *n* = 1; depression and anxiety (taking escitalopram and duloxetine for long term), *n* = 1.

^3^Urticaria, *n* = 1; asthma, *n* = 1; hypothyroidism (taking levothyroxine sodium for long term), *n* = 1.

^4^Compound vitamin.

**Supplemental Table 6.** **Moderate and** **vigorous physical activity during intervention periods^1^**

| Participant | Average time of moderate and vigorous physical activity per day, mean ± SD (min) | | | | | | *p* |
| --- | --- | --- | --- | --- | --- | --- | --- |
|  | Intervention periods | | | | | |  |
|  | 1 | 2 | 3 | 4 | 5 | 6 |  |
| *1* | 97.2±43.9 | 97.0±26.5 | 123±35.4 | 142±29.5 | 110±43.1 | 105±20.9 | 0.20 |
| *2* | 109±21.4 | 124±49.6 | 107±17.1 | 118±14.7 | 89.8±29.6 | 113±29.2 | 0.47 |
| *3* | 76.2±17.7 | 86.3±37.0 | 98.8±61.3 | 54.8±6.90 | 80.4±43.1 | 112±72.4 | 0.38 |
| *4* | 115±21.6 | 144±72.6 | 163±35.2 | 152±39.1 | 127±37.8 | 176±24.5 | 0.16 |
| *5* | 105±28.6 | NA^2^ | 90.6±37.2 | 77.8±37.6 | 87.6±49.4 | 70.0±22.9 | 0.60 |
| *6* | 144±8.60 | 133±17.8 | 149±26.4 | 122±22.8 | 116±25.4 | 158±39.4 | 0.05 |
| *7* | 116±50.2 | 68.5±15.3 | 119±55.5 | 140±84.7 | 128±74.4 | 108±65.4 | 0.47 |
| *8* | 126±10.4 | 139±47.0 | 157±36.0 | 123±19.2 | 125±22.8 | 123±21.5 | 0.28 |
| *9* | 128±16.4 | 101±20.5 | 104±24.6 | 112±39.1 | 127±41.0 | NA^2^ | 0.37 |
| *10* | 111±25.5 | 122±32.0 | 146±40.7 | 129±16.2 | 144±34.9 | 130±25.1 | 0.34 |
| *11* | 117±16.7 | 98.5±17.5 | 125±27.2 | 133±24.0 | 118±47.5 | 121±30.8 | 0.48 |
| *12* | 115±19.6 | 127±27.7 | 139±20.2 | 123±16.3 | 120±29.9 | 110±28.2 | 0.43 |
| *13* | 170±66.6 | 239±117.2 | 242±104 | 232±73.9 | 239±85.3 | 221±61.1 | 0.71 |
| *14* | 92.2±15.2 | 99.2±28.9 | 108±28.0 | 110±32.2 | 88.2±34.4 | 91.7±4.20 | 0.60 |
| *15* | 132±30.1 | 99.7±38.4 | 80.0±23.0 | 128±18.3 | 111±34.9 | 117±21.0 | 0.04 |
| *16* | 115±24.7 | 176±51.2 | 125±34.6 | 127±43.7 | 131±51.4 | 133±37.7 | 0.20 |
| *17* | 172±56.1 | 180±80.6 | 158±44.8 | 176±31.8 | 105±33.5 | 159±40.2 | 0.14 |
| *18* | 169±68.9 | 183±73.8 | 190±66.3 | 193±60.8 | 149±80.2 | 236±56.3 | 0.44 |
| *19* | 130±23.7 | 180±41.1 | 182±48.0 | 141±43.1 | 145±36.3 | 162±21.2 | 0.10 |
| *20* | 167±82.1 | 158±83.3 | 133±65.4 | 126±40.4 | 150±52.6 | 201±103 | 0.57 |
| *21* | 178±30.1 | NA^2^ | 156±30.7 | 168±50.3 | 169±33.9 | 176±46.7 | 0.90 |
| *22* | NA^2^ | 193±62.7 | 168±41.5 | 141±32.0 | 149±37.2 | 119±36.3 | 0.07 |
| *23* | 155±47.1 | 157±31.4 | 136±83.3 | 137±36.3 | 165±59.2 | 139±29.8 | 0.89 |
| *24* | 82.2±17.4 | 128±68.1 | 188±82.4 | 104±58.4 | 132±61.0 | 131±52.4 | 0.13 |
| *25* | 189±22.4 | 154±46.1 | 171±27.6 | 160±27.1 | 171±38.7 | 172±29.2 | 0.56 |
| *26* | 203±46.7 | 217±61.0 | 207±77.6 | 195±47.3 | 255±116 | 207±54.8 | 0.75 |
| *27* | 119±21.9 | 119±87.3 | 133±37.8 | 108±24.2 | 132±2.70 | 129±31.6 | 0.91 |
| *28* | 126±20.1 | 147±18.8 | 129±16.0 | 147±33.3 | 148±37.2 | NA^2^ | 0.43 |

^1^Values are means ± SDs, *n* = 6 days/period. Throughout each 6-d intervention period, all participants were asked to wear an AX3 band around the wrist for 24 hours/ day to monitor their physical activity of the whole day. AX3 band measured the physical activity intensity and output the value every minute. One-way ANOVA analysis was carried out on AX3 band data. Moderate physical activity refers to the physical activity that is performed between 3 and less than 6 times metabolic equivalent of tasks (METS). Vigorous physical activity refers to physical activity that is performed at 6.0 or more METS. Every intervention period lasted for 6 days.

^2^AX3 band failure and participants drop-out accounted for missing data in period 1 to 3 and the last period respectively.

**Supplemental Table 7. Individual data examples**^1^

| Set | Day | Participant *21*^2^ | | | | | | | Participant *8*^3^ | | | | | | |
| --- | --- | --- | --- | --- | --- | --- | --- | --- | --- | --- | --- | --- | --- | --- | --- |
|  |  | MPG (mmol/L) | | | MAGE (mmol/L) | | AUC_24_ (mmol/L·h) | | MPG (mmol/L) | | | MAGE (mmol/L) | | AUC_24_ (mmol/L·h) | |
|  |  | Meal^4^ | HF-LC | LF-HC | HF-LC | LF-HC | HF-LC | LF-HC | Meal^4^ | HF-LC | LF-HC | HF-LC | LF-HC | HF-LC | LF-HC |
| 1 | 1 | 1 | 8.50 | 8.20 | 1.58 | 2.10 | 124 | 126 | 1 | 7.40 | 9.10 | 2.00 | 2.50 | 102 | 97.9 |
|  |  | 2 | 6.40 | 6.90 |  |  |  |  | 2 | 5.30 | 4.70 |  |  |  |  |
|  |  | 3 | 6.20 | 6.00 |  |  |  |  | 3 | 5.70 | 5.20 |  |  |  |  |
| 1 | 2 | 1 | 8.60 | 8.00 | 2.67 | 1.86 | 122 | 124 | 1 | 8.40 | 7.50 | 3.20 | 4.40 | 115 | 110 |
|  |  | 2 | 6.20 | 6.40 |  |  |  |  | 2 | 5.30 | 5.50 |  |  |  |  |
|  |  | 3 | 7.10 | 6.20 |  |  |  |  | 3 | 7.20 | 8.20 |  |  |  |  |
| 1 | 3 | 1 | 8.30 | 7.10 | 1.38 | 1.18 | 119 | 119 | 1 | NA^5^ | 10.3 | 1.57 | 3.47 | 102 | 123 |
|  |  | 2 | 5.60 | 5.90 |  |  |  |  | 2 | NA^5^ | 6.0 |  |  |  |  |
|  |  | 3 | 5.90 | 5.90 |  |  |  |  | 3 | NA^5^ | 7.30 |  |  |  |  |
| 1 | 4 | 1 | 7.20 | 7.10 | 1.36 | 2.12 | 120 | 119 | 1 | 6.10 | 7.60 | 2.67 | 2.75 | 102 | 121 |
|  |  | 2 | 6.80 | 7.20 |  |  |  |  | 2 | 5.50 | 7.20 |  |  |  |  |
|  |  | 3 | 5.70 | 7.30 |  |  |  |  | 3 | 6.50 | 7.60 |  |  |  |  |
| 1 | 5 | 1 | 7.10 | 7.60 | 1.19 | 1.86 | 121 | 120 | 1 | 8.10 | 7.20 | 4.20 | 2.15 | 108 | 120 |
|  |  | 2 | 6.10 | 6.90 |  |  |  |  | 2 | 5.30 | 5.80 |  |  |  |  |
|  |  | 3 | 5.80 | 6.70 |  |  |  |  | 3 | 6.10 | 7.90 |  |  |  |  |
| 1 | 6 | 1 | 8.80 | 8.80 | 2.70 | 2.55 | 118 | 124 | 1 | 9.40 | 8.20 | 2.20 | 2.70 | 120 | 123 |
|  |  | 2 | 5.60 | 5.90 |  |  |  |  | 2 | 5.30 | 6.60 |  |  |  |  |
|  |  | 3 | 5.90 | 7.20 |  |  |  |  | 3 | 6.30 | 8.30 |  |  |  |  |
| 2 | 1 | 1 | 8.70 | 7.80 | 2.66 | 1.86 | 129 | 120 | 1 | 7.40 | 9.30 | 2.04 | 3.07 | 120 | 117 |
|  |  | 2 | 8.60 | 6.00 |  |  |  |  | 2 | 7.10 | 5.70 |  |  |  |  |
|  |  | 3 | 6.30 | 6.10 |  |  |  |  | 3 | 8.00 | 6.70 |  |  |  |  |
| 2 | 2 | 1 | 6.90 | 6.90 | 1.85 | 1.42 | 128 | 119 | 1 | 7.80 | 6.40 | 3.10 | 2.08 | 129 | 107 |
|  |  | 2 | 7.40 | 6.40 |  |  |  |  | 2 | 6.30 | 5.90 |  |  |  |  |
|  |  | 3 | 7.20 | 6.40 |  |  |  |  | 3 | 7.80 | 7.40 |  |  |  |  |
| 2 | 3 | 1 | 10.2 | 7.10 | 2.57 | 2.00 | 133 | 121 | 1 | 9.30 | 8.10 | NA^5^ | 1.82 | NA^5^ | 119 |
|  |  | 2 | 6.50 | 7.80 |  |  |  |  | 2 | 6.80 | 6.90 |  |  |  |  |
|  |  | 3 | 6.50 | 6.10 |  |  |  |  | 3 | 7.60 | 6.70 |  |  |  |  |
| 2 | 4 | 1 | 9.30 | 7.10 | 2.20 | 1.38 | 130 | 123 | 1 | 10.3 | 8.10 | 3.63 | 2.27 | 129 | 125 |
|  |  | 2 | 6.50 | 6.80 |  |  |  |  | 2 | 7.20 | 6.00 |  |  |  |  |
|  |  | 3 | 6.30 | 6.60 |  |  |  |  | 3 | 7.40 | 6.60 |  |  |  |  |
| 2 | 5 | 1 | 7.60 | 8.30 | 1.34 | 2.50 | 130 | 123 | 1 | 9.50 | 7.90 | 3.33 | 2.03 | 129 | 126 |
|  |  | 2 | 6.20 | 7.10 |  |  |  |  | 2 | 5.90 | 6.20 |  |  |  |  |
|  |  | 3 | 6.30 | 6.10 |  |  |  |  | 3 | 7.20 | 6.60 |  |  |  |  |
| 2 | 6 | 1 | 8.90 | 9.80 | 2.48 | 2.83 | 129 | 124 | 1 | 8.90 | 8.70 | 2.63 | 2.17 | 125 | 127 |
|  |  | 2 | 6.30 | 6.40 |  |  |  |  | 2 | 5.70 | 6.20 |  |  |  |  |
|  |  | 3 | 6.90 | 6.10 |  |  |  |  | 3 | 6.70 | 6.70 |  |  |  |  |
| 3 | 1 | 1 | 6.80 | 8.90 | 2.18 | 2.48 | 119 | 125 | 1 | 7.80 | 7.10 | 1.84 | 2.57 | 128 | 92 |
|  |  | 2 | 6.90 | 8.20 |  |  |  |  | 2 | 6.60 | 5.10 |  |  |  |  |
|  |  | 3 | 6.90 | 6.70 |  |  |  |  | 3 | 8.20 | 5.40 |  |  |  |  |
| 3 | 2 | 1 | 7.60 | 6.70 | 2.25 | 1.43 | 123 | 121 | 1 | 8.40 | 6.30 | 2.35 | 1.95 | 121 | 86.6 |
|  |  | 2 | 7.20 | 6.30 |  |  |  |  | 2 | 6.10 | 4.60 |  |  |  |  |
|  |  | 3 | 7.10 | 7.10 |  |  |  |  | 3 | 7.20 | 5.60 |  |  |  |  |
| 3 | 3 | 1 | 9.30 | 7.00 | 2.60 | 1.72 | 131 | 121 | 1 | 8.70 | 7.40 | 2.80 | 2.20 | 129 | 106 |
|  |  | 2 | 8.40 | 6.70 |  |  |  |  | 2 | 7.20 | 5.50 |  |  |  |  |
|  |  | 3 | 6.60 | 7.20 |  |  |  |  | 3 | 8.30 | 6.70 |  |  |  |  |
| 3 | 4 | 1 | 9.80 | 7.30 | 1.95 | 2.18 | 132 | 127 | 1 | 12.3 | 7.70 | 7.90 | 2.37 | 136 | 119 |
|  |  | 2 | 6.70 | 7.10 |  |  |  |  | 2 | 6.10 | 6.10 |  |  |  |  |
|  |  | 3 | 6.00 | 8.10 |  |  |  |  | 3 | 7.20 | 6.70 |  |  |  |  |
| 3 | 5 | 1 | 9.90 | 6.60 | 2.57 | 2.13 | 132 | 130 | 1 | 10.5 | 6.80 | 2.85 | 1.90 | 136 | 123 |
|  |  | 2 | 6.70 | 7.90 |  |  |  |  | 2 | 7.40 | 6.10 |  |  |  |  |
|  |  | 3 | 6.20 | 7.30 |  |  |  |  | 3 | 6.30 | 7.80 |  |  |  |  |
| 3 | 6 | 1 | 8.70 | 8.80 | 2.43 | 2.40 | 134 | 134 | 1 | 10.8 | 10.4 | 3.40 | 3.63 | 136 | 128 |
|  |  | 2 | 6.70 | 6.70 |  |  |  |  | 2 | 6.30 | 7.60 |  |  |  |  |
|  |  | 3 | 7.70 | 7.10 |  |  |  |  | 3 | 7.40 | 6.90 |  |  |  |  |

^1^Values are glucose responses based on continuous glucose monitors systems, *n* = 2. AUC_24_, area under the curve; HF-LC, high fat, low carbohydrate; LF-HC, low fat, high carbohydrate; MAGE, mean amplitude of glycemic excursions; MPG, maximum postprandial glucose.

^2^ Participant 21 was a non-responder.

^3^ Participant 8 was a high-fat responder for MPG and MAGE.

^4^ Meal 1, 2 and 3 represents breakfast, lunch and dinner respectively.

^5^Absence from intervention meals accounted for missing data.

**Supplemental Table 8. Mean difference between postprandial blood glucose** **when Chinese young adults consumed LF-HC compared with HF-LC diet**^1^

| Participant | MPG (mmol/L) | | MAGE (mmol/L) | | AUC_24_ (mmol/L·h) | |
| --- | --- | --- | --- | --- | --- | --- |
|  | Mean | 95% Credible interval | Mean | 95% Credible interval | Mean | 95% Credible interval |
| *1* | -0.106 | (-0.436, 0.200) | -0.267 | (-0.667, 0.116) | -0.123 | (-5.12, 4.89) |
| *2* | -0.0376 | (-0.412, 0.448) | 0.0402 | (-0.431, 0.515) | 1.27 | (-4.54, 7.14) |
| *3* | 0.304 | (-0.0455, 0.642) | -0.0473 | (-0.353, 0.262) | 3.13 | (-3.46, 9.81) |
| *4* | -0.0608 | (-0.545, 0.665) | -0.781 | (-1.77, 0.188) | -0.17 | (-5.28, 4.91) |
| *5* | 0.242 | (-0.177, 0.629) | 0.419 | (-0.0230, 0.865) | -3.05 | (-9.73, 3.71) |
| *6* | 0.391 | (0.00234, 0.759) | 0.169 | (-0.250, 0.592) | 5.95 | (1.77, 10.2) |
| *7* | 0.574 | (0.261, 0.889) | 0.0902 | (-0.155, 0.345) | 13.5 | (6.31, 21.0) |
| *8* | -0.411 | (-0.978, 0.113) | -0.474 | (-1.23, 0.280) | -6.09 | (-14.3, 2.19) |
| *9* | 0.192 | (-0.245, 0.625) | 0.100 | (-0.481, 0.711) | 0.314 | (-5.01, 5.91) |
| *10* | -0.0147 | (-0.409, 0.345) | 0.107 | (-0.264, 0.482) | -2.43 | (-7.37, 2.57) |
| *11* | -0.349 | (-0.708, 0.00881) | 0.0563 | (-0.326, 0.440) | -7.67 | (-13.1, -2.21) |
| *12* | -0.0139 | (-0.484, 0.464) | 0.0666 | (-0.626, 0.770) | -1.07 | (-6.94, 4.86) |
| *13* | -0.380 | (-0.785, 0.0112) | -0.223 | (-0.708, 0.282) | -2.71 | (-9.08, 3.75) |
| *14* | -0.224 | (-0.548, 0.0851) | 0.0368 | (-0.405, 0.482) | -4.59 | (-8.15, -1.00) |
| *15* | 0.280 | (-0.177, 0.728) | 0.240 | (-0.174, 0.657) | -0.76 | (-7.38, 5.94) |
| *16* | -0.173 | (-0.642, 0.306) | 0.285 | (-0.258, 0.856) | -11.1 | (-19.3, -3.26) |
| *17* | 0.298 | (-0.0237, 0.559) | 0.0205 | (-0.303, 0.346) | 2.87 | (-1.81, 7.61) |
| *18* | -0.264 | (-0.686, 0.140) | -0.239 | (-0.702, 0.221) | -1.23 | (-7.82, 5.40) |
| *19* | 0.916 | (0.501, 1.30) | 0.327 | (-0.0400, 0.688) | 15.2 | (7.23, 23.1) |
| *20* | 0.204 | (-0.297, 0.698) | -0.0759 | (-0.585, 0.459) | 3.87 | (-1.56, 9.60) |
| *21* | -0.121 | (-0.540, 0.261) | -0.103 | (-0.424, 0.222) | -2.89 | (-5.98, 0.236) |
| *22* | 0.806 | (0.347, 1.25) | 0.552 | (-0.0543, 1.16) | 10.5 | (6.77, 14.3) |
| *23* | 0.405 | (-0.00620, 0.806) | -0.148 | (-0.711, 0.395) | 10.2 | (-0.0954, 20.1) |
| *24* | -0.157 | (-0.472, 0.143) | -0.0206 | (-0.267, 0.242) | -3.83 | (-9.14, 1.27) |
| *25* | 0.319 | (-0.196, 0.789) | 0.327 | (-0.134, 0.791) | 3.54 | (-0.277, 7.40) |
| *26* | 0.284 | (-0.115, 0.700) | 0.182 | (-0.460, 0.794) | -1.73 | (-6.71, 3.03) |
| *27* | 0.378 | (-0.0167, 0.752) | 0.448 | (-0.0949, 0.971) | -1.05 | (-5.54, 3.29) |
| *28* | -0.316 | (-0.701, 0.0720) | -0.274 | (-0.606, 0.0748) | -4.30 | (-14.1, 6.03) |

^1^Values are means and 95% credible intervals, *n* = 28. AUC_24_, area under the curve; HF-LC, high fat, low carbohydrate; LF-HC, low fat, high carbohydrate; MAGE, mean amplitude of glycemic excursions; MPG, maximum postprandial glucose.

**Supplemental Table 9. Results from Bayesian analysis at the group level** **when Chinese young adults consumed LF-HC compared with HF-LC diet**^1^

| Participants included in analysis | MPG (mmol/L) | | | MAGE (mmol/L) | | | AUC_24_ (mmol/L·h) | | |
| --- | --- | --- | --- | --- | --- | --- | --- | --- | --- |
|  | Mean difference^2^ | 95% Credible interval of the difference | Posterior Probability^3^ (%) | Mean difference^2^ | 95% Credible interval of the difference | Posterior Probability^3^ (%) | Mean difference^2^ | 95% Credible interval of the difference | Posterior Probability^3^ (%) |
| *28* | 0.0866 | (-0.0443, 0.221) | 11.8 | 0.0335 | (-0.0762, 0.144) | 27.0 | 0.406 | (-1.51, 2.30) | 0 |
| *26* | 0.108 | (-0.0337, 0.250) | 20.1 | 0.0388 | (-0.0750, 0.153) | 30.7 | 0.546 | (-1.66, 2.59) | 0 |
| *25* | 0.108 | (-0.0346, 0.249) | 19.5 | 0.0324 | (-0.103, 0.164) | 32.2 | 0.648 | (-1.74, 2.96) | 0 |
| *21* | 0.110 | (-0.0659, 0.289) | 25.2 | 0.0589 | (-0.0728, 0.195) | 44.7 | 1.086 | (-1.51, 3.84) | 0 |

^1^Values are means, 95% credible intervals and posterior probabilities, *n* = 28 (all available data), 26 (excluding participants who withdrew early from the trial), 25 (excluding those who withdrew early and those with valid data less than 50%) or 21 (individuals who completed the entire protocol with neither chronic diseases nor taking prescription medications). A Bayesian hierarchical model was applied to aggregate the data and noninformative priors were employed. Specifically, we specified a generalized linear mixed regression (adjusted for age, sex and BMI), which constructed a separate regression model incorporating serial correlation that related longitudinal measurements for each participant and assuming a normal distribution of the measurements centered around each participant’s true intervention effect. These regression models were then connected through a second-level random effects model which postulated that the subject-specific regression coefficients were related through a normal distribution centered around the population-level average coefficients. The variability of these random effects represents the variability in the inter-individual treatment effects. AUC_24_, area under the curve; MAGE, mean amplitude of glycemic excursions; MPG, maximum postprandial glucose.

^2^The difference between MPG elicited by low fat, high carbohydrate diet and high fat, low carbohydrate diet.

^3^The posterior probability of reaching clinical meaningful difference (MPG > 0.167 mmol/L or < -0.167 mmol/L; MAGE > 0.072 mmol/L or < -0.072 mmol/L; AUC_24_ > 13.889 mmol/L or < -13.889 mmol/L).

**Supplemental Figure 1. Study Design and Flow Diagram**


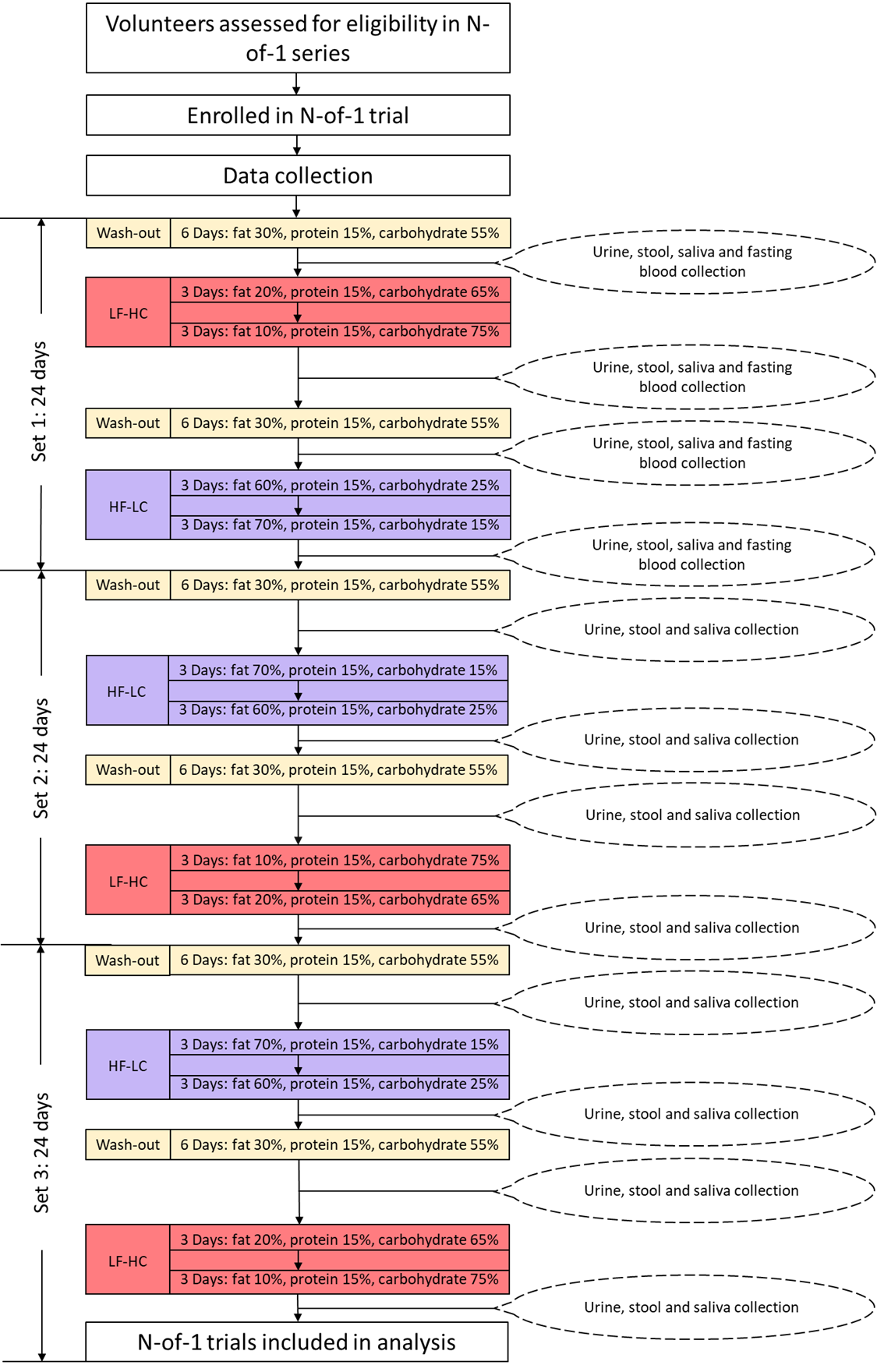


The sequence of each diet was randomized. Urine and stool were collected on the first day of each wash-out and intervention period throughout the trial; fasting venous blood was collected on the first day of each wash-out and intervention period in set 1. Participants *n* = 28. HF-LC, high fat, low carbohydrate; LF-HC, low fat, high carbohydrate.

**Supplemental Figure 2. Effect by macronutrient categorization for the difference in the carbohydrate/fat intake with the postprandial blood glucose response.**

**
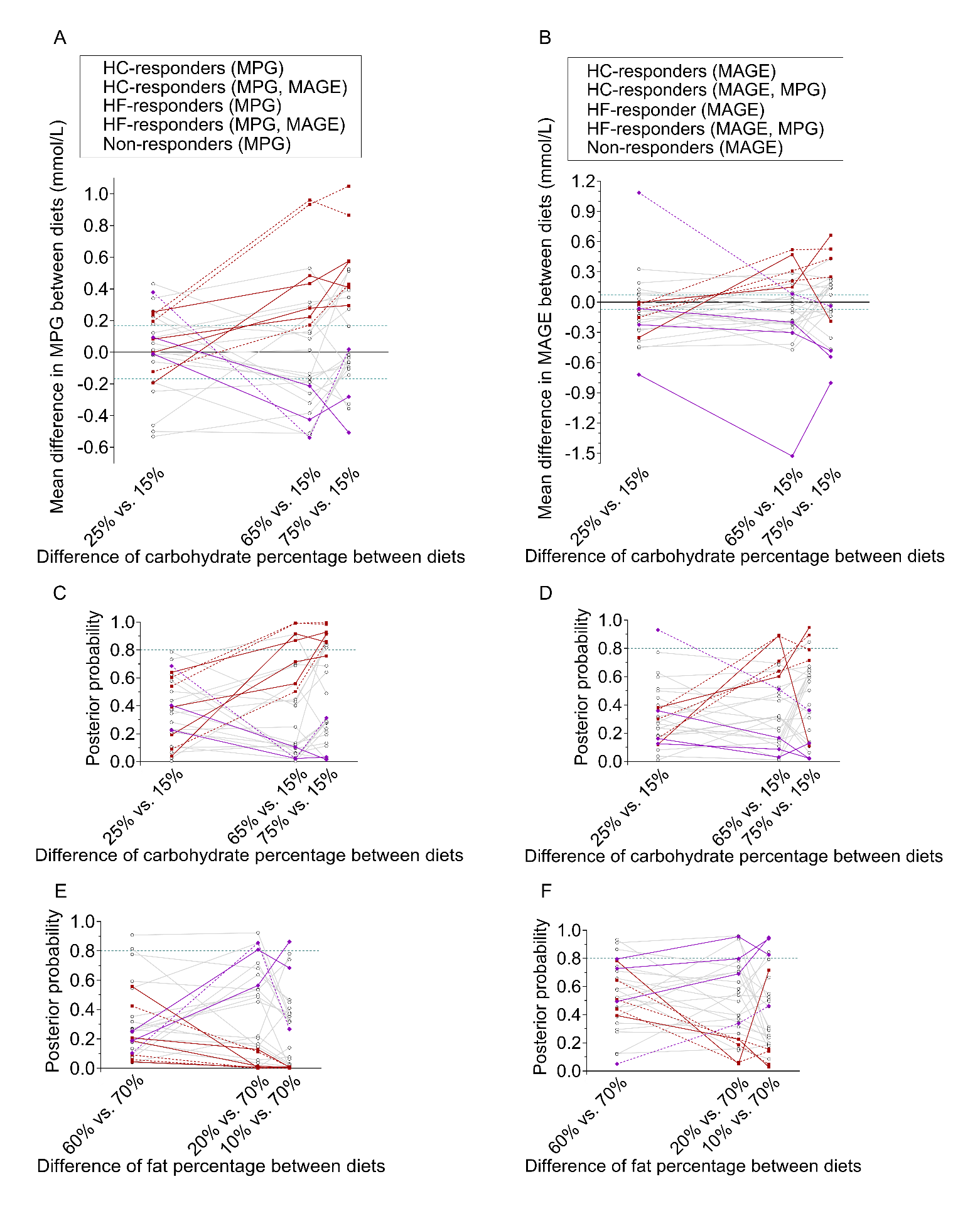
**

For the three groups of points on the x-axis, the difference of energy proportion from carbohydrate is 10%, 50% and 60% respectively relative to the diet with lowest carbohydrate content in the trial. Bayesian analysis was performed between the three paired diets, 25% vs. 15% carbohydrate diet, 65% vs. 15% carbohydrate diet, and 75% vs. 15% carbohydrate diet. **(A)** The y-axis indicates the mean difference between maximum postprandial glucose (MPG) from LF-HC and HF-LC diets, *n* = 28. Individual-level effects are ordered from smallest to largest probability on the y-axis for the three macronutrients difference. Squares, rhombus and open circles indicate high-carbohydrate responders (MPG) (*n* = 7), high-fat responders (MPG) (*n* = 3) and non-responders (MPG) (*n* = 18) respectively. Each spot connected by a line represents an individual (*n* = 28) in the trial and dashed lines connect those responders for MPG as well as mean amplitude of glycemic excursions (MAGE) (*n* = 3). Green dotted lines at y = 0.167 and -0.167 represent the threshold for a clinically meaningful effect. **(B)** The y-axis indicates the mean difference between MAGE from LF-HC and HF-LC diets, *n* = 28. Green dotted lines at y = 0.072 and -0.072 represent the threshold for a clinically meaningful effect. **(C)** The y-axis indicates the posterior probability of difference between MPG from each paired diet higher than 0.167 mmol/L, *n* = 28. Green dotted line at y = 0.8 represents the threshold for responders. **(D)** The y-axis indicates the posterior probability of difference between MAGE from each paired diet higher than 0.072 mmol/L, *n* = 28. **(E)** The y-axis indicates the posterior probability of difference between MPG from each paired diet lower than -0.167 mmol/L, *n* = 28. **(F)** The y-axis indicates the posterior probability of difference between MAGE from each paired diet lower than -0.072 mmol/L, *n* = 28. HF-LC, high-fat low-carbohydrate; LF-HC, low-fat high-carbohydrate; MAGE, mean amplitude of glycemic excursions; MPG, maximum postprandial glucose.
